# The Menstrual Cycle Modulates Whole-Brain Turbulent Dynamics

**DOI:** 10.3389/fnins.2021.753820

**Published:** 2021-12-09

**Authors:** Eleonora De Filippi, Carme Uribe, Daniela S. Avila-Varela, Noelia Martínez-Molina, Venera Gashaj, Laura Pritschet, Tyler Santander, Emily G. Jacobs, Morten L. Kringelbach, Yonatan Sanz Perl, Gustavo Deco, Anira Escrichs

**Affiliations:** ^1^Computational Neuroscience Group, Center for Brain and Cognition, Department of Information and Communication Technologies, Universitat Pompeu Fabra, Barcelona, Spain; ^2^Research Imaging Centre, Campbell Family Mental Health Research Institute, Centre for Addiction and Mental Health (CAMH), University of Toronto, Toronto, ON, Canada; ^3^Medical Psychology Unit, Department of Medicine, Institute of Neuroscience, University of Barcelona, Barcelona, Spain; ^4^Speech Acquisition and Perception Group, Center for Brain and Cognition, Universitat Pompeu Fabra, Barcelona, Spain; ^5^Swiss Federal Institute of Technology (ETH), Zurich, Switzerland; ^6^Department of Psychological and Brain Sciences, University of California, Santa Barbara, Santa Barbara, CA, United States; ^7^Neuroscience Research Institute, University of California, Santa Barbara, Santa Barbara, CA, United States; ^8^Centre for Eudaimonia and Human Flourishing, University of Oxford, Oxford, United Kingdom; ^9^Department of Psychiatry, University of Oxford, Oxford, United Kingdom; ^10^Department of Clinical Medicine, Center for Music in the Brain, Aarhus University, Aarhus, Denmark; ^11^Institució Catalana de la Recerca i Estudis Avançats (ICREA), Barcelona, Spain; ^12^Department of Neuropsychology, Max Planck Institute for Human Cognitive and Brain Sciences, Leipzig, Germany; ^13^Turner Institute for Brain and Mental Health, Monash University, Melbourne, VIC, Australia

**Keywords:** menstrual cycle, turbulence, brain information processing, whole-brain dynamics, resting-state fMRI

## Abstract

Brain dynamics have recently been shown to be modulated by rhythmic changes in female sex hormone concentrations across an entire menstrual cycle. However, many questions remain regarding the specific differences in information processing across spacetime between the two main follicular and luteal phases in the menstrual cycle. Using a novel turbulent dynamic framework, we studied whole-brain information processing across spacetime scales (i.e., across long and short distances in the brain) in two open-source, dense-sampled resting-state datasets. A healthy naturally cycling woman in her early twenties was scanned over 30 consecutive days during a naturally occurring menstrual cycle and under a hormonal contraceptive regime. Our results indicated that the luteal phase is characterized by significantly higher information transmission across spatial scales than the follicular phase. Furthermore, we found significant differences in turbulence levels between the two phases in brain regions belonging to the default mode, salience/ventral attention, somatomotor, control, and dorsal attention networks. Finally, we found that changes in estradiol and progesterone concentrations modulate whole-brain turbulent dynamics in long distances. In contrast, we reported no significant differences in information processing measures between the active and placebo phases in the hormonal contraceptive study. Overall, the results demonstrate that the turbulence framework is able to capture differences in whole-brain turbulent dynamics related to ovarian hormones and menstrual cycle stages.

## 1. Introduction

The brain is one of the most complex systems, and it is intrinsically modulated by sex steroid hormone fluctuations (for a review, see Beltz and Moser, 2020). Ovarian hormones represent the neuroendocrine milieu throughout the female lifespan (McEwen et al., 2012). For instance, previous studies have demonstrated the significant influence of estradiol and progesterone in cognitive, emotional and social functioning (Toffoletto et al., 2014; Barth et al., 2015; Galea et al., 2017). Estradiol and progesterone concentrations are related to hippocampal connectivity, upholding memory retrieval (Jacobs et al., 2016, 2017). Psychosocial stress response seems to be also modulated by estradiol as high concentrations are related to deactivation of the limbic system in contrast with lower estradiol levels (Albert et al., 2015). In naturally-cycling women, a typical menstrual cycle occurs every 25–32 days (Lenton et al., 1984, for a meta-review, see Fehring et al., 2006) and comprises two main broad phases, the follicular and the luteal phases (Guyton and Hall, 2006; Song et al., 2017; Garcia et al., 2018; Olatunji et al., 2020; Schmalenberger et al., 2020). The follicular phase begins with the onset of menses, and it is characterized by a progressive increase in estradiol concentrations that reach the maximum peak during the pre-ovulatory phase, during which progesterone levels are at their lowest. In contrast, the luteal phase, which spans after ovulation occurs until the last day of the menstrual cycle, is marked by an increment in progesterone levels that reach their peak near the middle of the phase, when estradiol typically experiences a secondary mid-luteal peak (Reed and Carr, 2000; Bull et al., 2019).

Exploring differences in the brain as a function of the menstrual cycle phases and ovarian hormone levels has been undertaken using several imaging modalities (Witte et al., 2010; Jacobs and D'Esposito, 2011; Rapkin et al., 2011; Petersen et al., 2014; Arélin et al., 2015; Lisofsky et al., 2015b; Catenaccio et al., 2016; Engman et al., 2018; Weis et al., 2019; for a review, see Dubol et al., 2021). To date, human brain imaging studies have typically sampled women across a limited set of days, which do not represent the whole rhythmic variability of hormone production over an entire menstrual cycle. More recently, a dense-sampling neuroimaging study has shown positive associations between estradiol and dynamic, spatially-diffuse changes in resting-state networks from a 30 consecutive days assessment of a young woman, thus enabling the study of functional connectivity over a complete menstrual cycle (Pritschet et al., 2020; Mueller et al., 2021).

Yet, the effects of the two main phases of the menstrual cycle and day-to-day hormonal changes on whole-brain information processing remain unclear. Recently, a novel framework has been proposed to describe information processing at the macroscale level by demonstrating the presence of turbulent dynamics in the human brain (Deco and Kringelbach, 2020; Deco et al., 2021a). Turbulent brain dynamics have been explored in a large sample of healthy subjects, where enhanced transmission of information across the whole brain was determined by the local synchronization between brain regions (Deco and Kringelbach, 2020). Inspired by the advances in corroborating the presence of turbulent behavior in fluid dynamics (Kuramoto, 1984; Kolmogorov, 1991a,b), this framework has sought to demonstrate the link between this level of local synchronization among brain areas with the rotational vortices found in fluid dynamics. Therefore, the novelty of the method is that it allows analyzing the brain's information processing across spacetime scales, given that the size of rotational vortices determines various scales of information transmission. This framework has been successfully applied to characterize turbulent behavior in the brain's information processing during rest and different cognitive tasks (Deco and Kringelbach, 2020), demonstrated that different levels of turbulent dynamics describe and differentiate between unconscious and conscious brain states (Escrichs et al., 2021), and showed how large-scale connections enhance the transmission of information across the whole-brain network (Deco et al., 2021b). Therefore, the turbulent framework may advance our understanding of the effect of the two main phases of the menstrual cycle (i.e., follicular and luteal) on large-scale brain network communication.

Here, we applied the turbulent framework (Deco and Kringelbach, 2020; Deco et al., 2021b; Escrichs et al., 2021) to the same deep-sampling datasets used in Pritschet et al. (2020) and Mueller et al. (2021) to investigate the brain's information processing across spacetime scales during the follicular and luteal phases of a woman's menstrual cycle. First, we applied a turbulent dynamic analysis while the participant, scanned daily over an entire menstrual cycle (*N* = 30 days), was freely cycling (NaturalMenstrualCycle Study). In addition, we investigated turbulent dynamics while the same participant was under a hormonal-based regime to compare differential endocrine states on the brain's information processing (HormonalContraceptive Study). Moreover, we computed multi-level models with measures of information processing as output, and estradiol and progesterone as predictors to understand the relationship between day-by-day ovarian hormone levels and whole-brain turbulent dynamics.

## 2. Methods

### 2.1. Participant and Study Design

A healthy woman was scanned during 30 consecutive days over a complete natural menstrual cycle and while taking a hormonal contraceptive pill (aged 23 and 24, respectively). The participant underwent time-locked blood sampling to determine hormone levels before each MRI session. The participant had no history of psychiatric nor endocrine disorders. During the NaturalMenstrualCycle Study, the participant was naturally-cycling and had not undergone any hormonal treatment 12 months before the study. In the HormonalContraceptive Study, the subject was under a hormonal-based regime (0.02 mg ethinylestradiol, 0.1 mg levonorgestrel, Aubra, Afaxys Pharmaceuticals) for 10 months before acquiring the data. This hormonal regime selectively suppressed progesterone levels, allowing estradiol to fluctuate at comparable levels to the NaturalMenstrualCycle Study. For further information about the dataset, we refer readers to Pritschet et al. (2020) and Mueller et al. (2021).

### 2.2. MRI Data Acquisition

Raw data of daily scanning for both studies were obtained from the open-access project “28andMe” (https://openneuro.org/datasets/ds002674/versions/1.0.5). Anatomical and functional data were acquired on a Siemens 3T Prisma scanner with a 64-channel phased-array head coil. High-resolution T1-weighted images were acquired with magnetization prepared rapid gradient echo (MPRAGE) sequence (TR = 2,500 ms, TE = 2.31 ms, TI = 934 ms, flip angle = 7°, 0.8 mm thickness) with a gradient echo fieldmap (TR = 758 ms; TE1 = 4.92 ms; TE2 = 7.38 ms; flip angle = 60°). Resting-state data were acquired using a T2^*^-weighted multi-band echo-planar imaging (EPI) sequence (72 oblique slices, TR = 720 ms, TE = 37 ms, voxel size=2*mm*^3^, flip angle = 56°, multiband factor = 8) for a total of 820 volumes (=10 min). A complete description of the MRI acquisition can be consulted in Pritschet et al. (2020) and Mueller et al. (2021).

### 2.3. Resting-State fMRI Preprocessing

We computed preprocessing of functional data using the Data Processing Assistant for Resting-State fMRI (DPARSF) (Yan and Zang, 2010). Before preprocessing, functional and anatomical images were manually reoriented. Then, resting-state data were corrected for differences in slice acquisition time, and the first 5 time-points were discarded to allow for signal stabilization. Further preprocessing steps included realignment for motion correction across volumes and co-registration of functional images to T1-weighted images using unified segmentation. Then, a regression of nuisance covariates was applied to correct data for six head movement parameters, for global mean signal, the white matter, and cerebrospinal fluid signal. Subsequent steps included spatial normalization in MNI space, smoothing using a 6-mm full-width-at-half-maximum Gaussian kernel, and band-pass temporal filtering of 0.01–0.1 Hz. Lastly, time series were extracted using the Schaefer parcellation comprising 1,000 regions and 17 resting-state networks (Schaefer et al., 2018).

### 2.4. Probabilistic Tractography Analysis

We computed the T2-weighted and diffusion spectrum images of 32 participants from the Human Connectome Project (HCP) as reported in Deco and Kringelbach (2020). The acquisition parameters are described in detail on the HCP website (Setsompop et al., 2013). The openly Lead-DBS software (https://www.lead-dbs.org/) provides the preprocessing described in Horn et al. (2017). In brief, data were pre-processed using a q-sampling imaging algorithm performed in DSI studio (http://dsi-studio.labsolver.org). A white-matter mask was computed by segmenting the T2-weighted anatomical images and co-registering the images to the b0 of the diffusion MRI data by using SPM12. For each participant, 200,000 fibers were sampled within the white-matter mask. Fibers were converted to MNI space using Lead-DBS (Horn and Blankenburg, 2016). Finally, we applied the methods in Lead-DBS to obtain the structural connectomes from the Schaefer 1,000 parcellation (Schaefer et al., 2018).

### 2.5. Turbulent Framework

**Figure 1:**
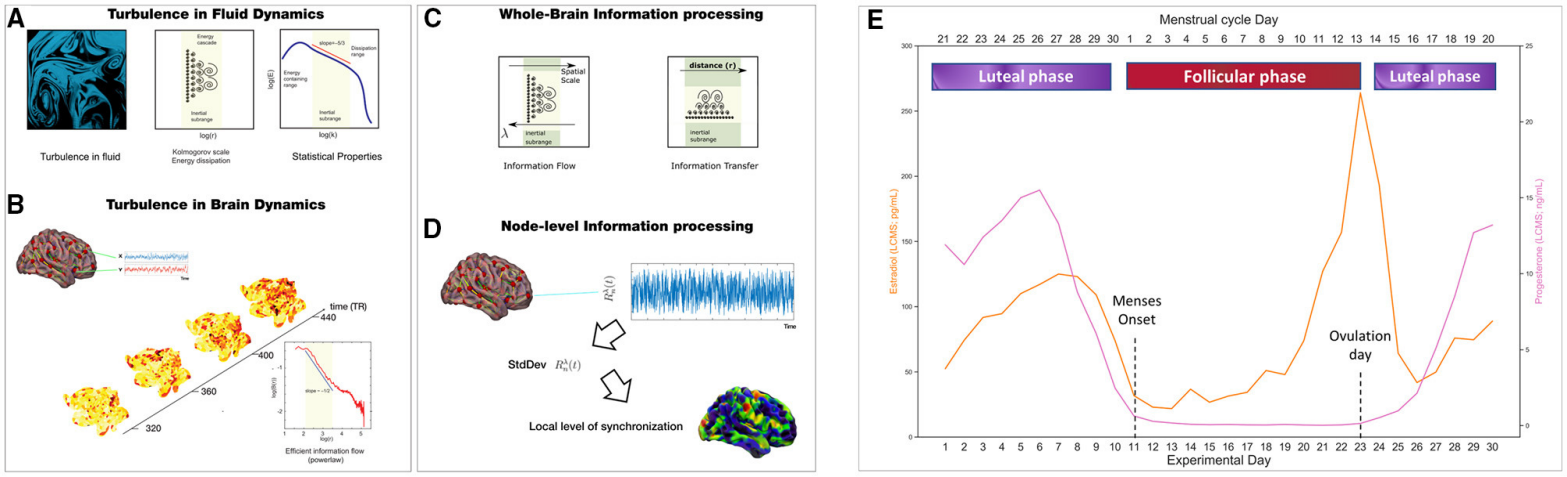
Methods: Turbulent framework and menstrual cycle parsing into phases. We studied how the brain information processing during resting-state varies between the follicular (red) and luteal (purple) phases of a woman menstrual cycle by applying the Turbulent Framework (Deco and Kringelbach, 2020). We computed 4 measures: the level of amplitude turbulence, information cascade flow, information cascade, and information transfer. **(A)** These measurements are based on Kolmogorov's studies demonstrating the presence of turbulence in fluid dynamics. **(B)** More recently, turbulent dynamics have been found in the human brain, for which the level of amplitude turbulence can be calculated. **(C)** Information cascade flow and information transfer are two measurements of information transmission across the whole-brain at different spatial scales by taking into account the Euclidean distance between brain areas. **(D)** This panel shows how turbulence levels, which reflect information processing capability, can be calculated at the node-level to understand which brain areas show the highest values in node-level information processing. **(E)** The lower x-axis shows the days of the experiment (1-30), whereas the upper x-axis indicates the division of the menstrual cycle phases. The phases were divided as follows: Follicular phase (days of the experiment: 11 to 23, where 11 is the onset of menses and 23 is the day of ovulation). The luteal phase included the days after ovulation (days of the experiment: 24-30) and days before menses (days of the experiment: 1-10). For 30 consecutive days, the subject provided blood samples to examine serum hormone concentrations (Estradiol, orange line in pg/mL; Progesterone, purple line in ng/mL). LC-MS, Liquid Chromatography-Mass Spectrometry. **(A–D)** adapted from Deco and Kringelbach (2020), Deco et al. (2021a) and Escrichs et al. (2021)

To understand the whole-brain turbulent dynamics underlying the menstrual cycle, we applied the turbulent framework (Deco and Kringelbach, 2020; Deco et al., 2021b). We investigated information processing across spacetime scales (i.e., over long and short distances in the brain) between the two main phases (i.e., follicular and luteal) of the participant's menstrual cycle. In particular, the information processing measures (Figures 1A–D) are based on Kuramoto's studies of coupled oscillators describing turbulent fluid dynamics (Kuramoto, 1984). We computed four empirical measures for each condition using a range of five spatial scales, λ, namely λ = 0.24, λ = 0.21, λ = 0.18, λ = 0.15 and λ = 0.12, where higher λ values represent shorter distances in the brain, and lower λ values reflect long distances. Moreover, we computed the same empirical measures for a second dataset of the same woman undergoing hormonal treatment as a comparison between hormonal states. A complete description of the methods can be consulted in Deco and Kringelbach (2020) and Deco et al. (2021b).

#### 2.5.1. Kuramoto Local Order Parameter

We computed the amplitude turbulence, $R_{\lambda }\left(\bar{x},t\right)$, as the modulus of the Kuramoto local order parameter for a given node as a function of time as follows:


(1) $$\begin{array}{l} R_{\lambda }\left(\bar{x},t\right)e^{i\vartheta_{\lambda }\left(\bar{x},t\right)}=k\int_{-\infty }^{\infty }d\bar{x}\prime G_{\lambda }\left(\bar{x}-{\bar{x}}^{\prime }\right)e^{i\varphi \left({\bar{x}}^{\prime },t\right)} \end{array}$$

where $\varphi \left(\bar{x},t\right)$ represents the phases of the BOLD signal data, *G*_λ_ refers to the local weighting kernel $G_{\lambda }\left(\bar{x}\right)=e^{-\lambda |\bar{x}|}$, *k* represents the normalization factor $\left[\int_{-\infty }^{\infty }{d\bar{x}\prime G_{\lambda }\left(\bar{x}-{\bar{x}}^{\prime }\right)}^{-1}\right]$, and λ defines the spatial scaling.

#### 2.5.2. Amplitude Turbulence

The level of amplitude turbulence is represented by the standard deviation of the modulus of the Kuramoto local order parameter (*R*_λ_):


(2) $$\begin{array}{l} D=<{\left(R_{\lambda }\right)}^{2}{>}_{\left(n,t\right)}-<R_{\lambda }{>}_{\left(n,t\right)}^{2} \end{array}$$

and the brackets < >_(*n,t*)_ represent average values across nodes and time.

#### 2.5.3. Information Cascade Flow and Information Cascade

The information cascade flow is computed as the time correlation between the Kuramoto local order parameter in two consecutive scales and time points:


(3) $$\begin{array}{l} IF\left(\lambda \right)=<corr_{t}\left(R_{\lambda }\left(\bar{x},t+\Delta t\right),R_{\lambda -\Delta \lambda }\left(\bar{x},t\right)\right){>}_{n} \end{array}$$

This equation describes how information is transmitted across different scales λ − Δλ, where Δλ represents the scale step in consecutive time steps (*t* and *t* + Δ*t*) and the brackets < >_*n*_ represent average values across nodes.

Then, the information cascade is computed by averaging the information cascade flow across scales λ, thus capturing the whole information processing profile across all scales.

#### 2.5.4. Information Transfer

The spatial information transfer describes how information is transmitted across the whole-brain at a given scale (i.e., λ) and is computed as the slope of a linear fitting in a log-log scale of the time correlation between the Kuramoto local order parameter in pairs of nodes as a function of the Euclidean distance (*r*) within the inertial subrange:


(4) $$\begin{array}{l} log\left(corr_{t}\left(R_{n}^{\lambda },R_{p}^{\lambda }\right)\left(r\right)\right)=A*log\left(r\right)+B \end{array}$$

*A* and *B* are the fitting parameters and *A* (i.e., the negative slope) represents the spatial information transfer.

#### 2.5.5. Node Variability of Local Synchronization

The node variability, NVLS, of the local synchronization is defined as standard deviation across time of the local Kuramoto order parameter as:


(5) $$\begin{array}{l} NVLS\left(n\right)=<R_{n}^{\lambda }{\left(t\right)}^{2}{>}_{t}-<R_{n}^{\lambda }\left(t\right){>}_{t}^{2} \end{array}$$

where brackets < >_*t*_ represent average values across time.

We estimated the discrete version of the node-level Kuramoto order parameter, with modulus *R* and phase ν, which represents a spatial average of the complex phase factor of the local oscillators weighted by the coupling, measured as:


(6) $$\begin{array}{l} R_{n}^{\lambda }\left(t\right){\text{ e}}^{\text{i}{\text{ν}}_{\text{n}}\left(\text{t}\right)}=\underset{p}{\sum }\left[\frac{{\text{C}}_{\text{np}}^{\text{λ}}}{\sum_{\text{q}}\;C_{\text{nq}}^{\text{λ}}}\right]{\text{e}}^{\text{i}{\text{φ}}_{\text{p}}\left(\text{t}\right)} \end{array}$$

where φ_*p*_(*t*) represents the phases of the BOLD signal data, and $C_{nq}^{\lambda }$ refers to the local weighting kernel between nodes *n* and *p*:


(7) $$\begin{array}{l} C_{np}={\text{e}}^{-\lambda \left(r\left(n,p\right)\right)} \end{array}$$

where *r*(*n, p*) is the Euclidean distance between nodes *n* and *p* in MNI space, and λ is the scaling of the local weighting obtained by adjusting the structural connectome matrix.

### 2.6. Statistical Analysis

We investigated the two main phases of the menstrual cycle (i.e., follicular and luteal). To this end, we divided the phases for the NaturalMenstrualCycle Study as follows: Follicular phase (days of the experiment: 11–23, where 11 is the onset of menses and 23 is the ovulation day). In the luteal phase, we included the days after ovulation (days of the experiment: 24–30) and days before menses (days of the experiment: 1–10). Please see Figure 1E. This division follows regular standards to divide the menstrual cycle into two phases (Olatunji et al., 2020; Schmalenberger et al., 2020). For statistical comparisons, we applied the Wilcoxon rank-sum method to test the differences between phases. We also performed group analyses removing the ovulation window (days of the experiment 12–14), which is characterized by a strong peak in estradiol levels (Pritschet et al., 2020). Concerning the HormonalContraceptive Study, we tested differences when the participant took exogenous hormonal contraceptive pills (active condition) and placebo inactive pills (placebo condition). Finally, to understand the relationship between changes in hormone concentrations and turbulent brain dynamics, we carried out multi-level models for each dependent variable (brain information processing measures) with hormone levels (estradiol and progesterone) as fixed effects and participant and session day of testing as random effects.

## 3. Results

**Figure 2:**
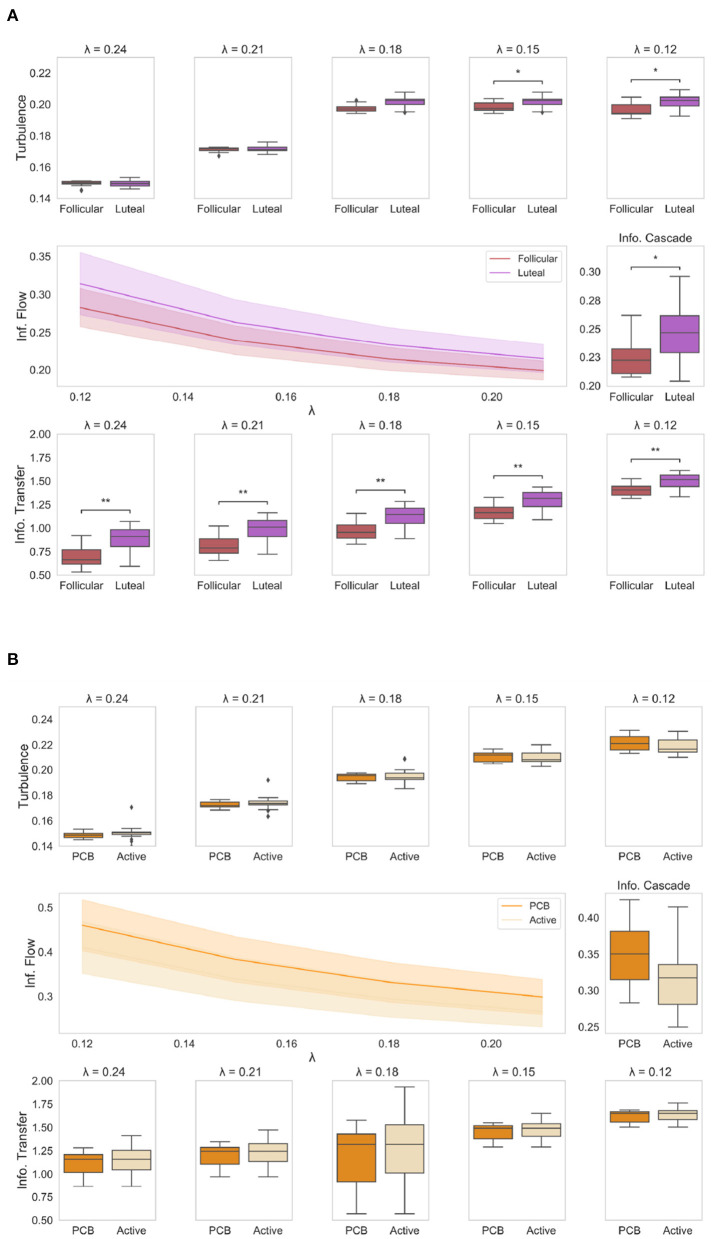
Results of information processing analysis: **(A)** The boxplots show the level of amplitude turbulence between the two phases of the NaturalMenstrualCycle Study (red: follicular, purple: luteal) across different spatial scales (i.e., *lambda*). We show that the turbulence level (upper plots) is significantly higher in lower scales (i.e., long distances) in the luteal phase than in the follicular (*p* < 0.05). The plot in the middle shows how the luteal phase (purple line) is characterized by higher information cascade flow across all scales than the follicular phase (red line). This is clearly displayed in the middle boxplot representing a higher average value of information cascade flow across scales (i.e., information cascade) for the luteal phase compared to the follicular (*p* < 0.05). Similarly, the boxplots at the bottom of the figure show how the information transfer is significantly reduced in the follicular phase compared to the luteal across all scales (*p* < 0.001). **(B)** The boxplots represent the four empirical measures (amplitude turbulence at the top; information cascade flow and information cascade in the middle; information transfer at the bottom) for HormonalContraceptive Study, comparing placebo (dark orange) and active (light orange) phases. We show that there is no difference in any of the four measures under the Turbulence framework between the active and placebo phases when the participant is under a hormone-based regime. *represents *p* < 0.05 while ** represents *p* < 0.01.

Results of information processing analysis are presented in Figure 2.

### 3.1. Amplitude Turbulence

First, we computed the amplitude turbulence across different spatial scales, which is defined as the standard deviation of the modulus the local Kuramoto order parameter applied to the empirical functional data (Kawamura et al., 2007). We found that the amplitude turbulence level (Figure 2A, upper panel) was significantly higher for the luteal phase compared to the follicular (*p* < 0.05) in lower values of λ, i.e., in long distances. In particular, we found significant higher levels of amplitude turbulence in the luteal phase at λ = 0.15 (*p* = 0.01) and λ = 0.12 (*p* = 0.01), whereas differences between follicular and luteal phases were not significant at λ=0.24, λ = 0.21 and λ = 0.18 (*p* > 0.05). This suggests that when naturally-cycling, the luteal phase is characterized by higher levels of amplitude turbulence across long distances. In contrast, we did not find any significant difference between the active and placebo conditions of the HormonalContraceptive Study (Figure 2B, upper panel) at any scale (*p* > 0.05).

### 3.2. Information Cascade Flow and Information Cascade

Next, we calculated information cascade flow, which indicates how information is transmitted over time from one given scale to another. This measure is defined as the time correlation between the Kuramoto order parameter into two sequential spatial scales. Then, to understand the efficiency of information transmission, we computed the information cascade by averaging the information cascade flow across all λ scales. The information cascade flow and information cascade results for each condition are presented in Figure 2, middle panel. The information cascade flow (Figure 2A, left panel) was significantly higher in the luteal phase compared to the follicular (*p* < 0.05) across all scales. The information cascade (Figure 2A middle, right panel) shows significantly higher values for the luteal phase compared to the follicular (*p* = 0.04). This result indicates that the information transmission across scales is enhanced in the luteal phase compared to the follicular phase. By contrast, we reported no significant differences in information cascade flow and information cascade between the active and placebo phases of the HormonalContraceptive Study (*p* > 0.05). Together, our results show that being on a hormonal contraceptive regime leads to a more stable information transmission pattern across the menstrual cycle, while during naturally cycling, information transmission is enhanced across the whole-brain network in the luteal phase.

### 3.3. Information Transfer

Lastly, we calculated the information transfer (Figure 2A, lower panel), which reflects how information travels across space at a particular scale, λ, computed as the slopes in the decay of the information transmission. The information transfer was significantly reduced during the follicular phase compared to the luteal at all λ scales (*p* < 0.05), meaning that the luteal phase leads to better information transfer than the follicular phase across all scales. Interestingly, being on a hormone-based regime appeared to cancel this effect with active and placebo phases showing similar levels of information transfer across all spatial scales (*p* > 0.05 for all λ values, Figure 2B, lower panel).

Remarkably, removal of the ovulation window in the NaturalMenstrualCycle Study did not change the results of any of the four measures. To compare the consistency of our findings, we also performed the analysis of the HormonalContraceptive Study by splitting it up into a simulated “follicular” and “luteal” phases as in NaturalMenstrualCycle Study. Once more, we found no differences between the two phases in the HormonalContraceptive Study for any of the four measures (*p* > 0.05).

### 3.4. Node Variability of Local Synchronization

**Figure 3:**
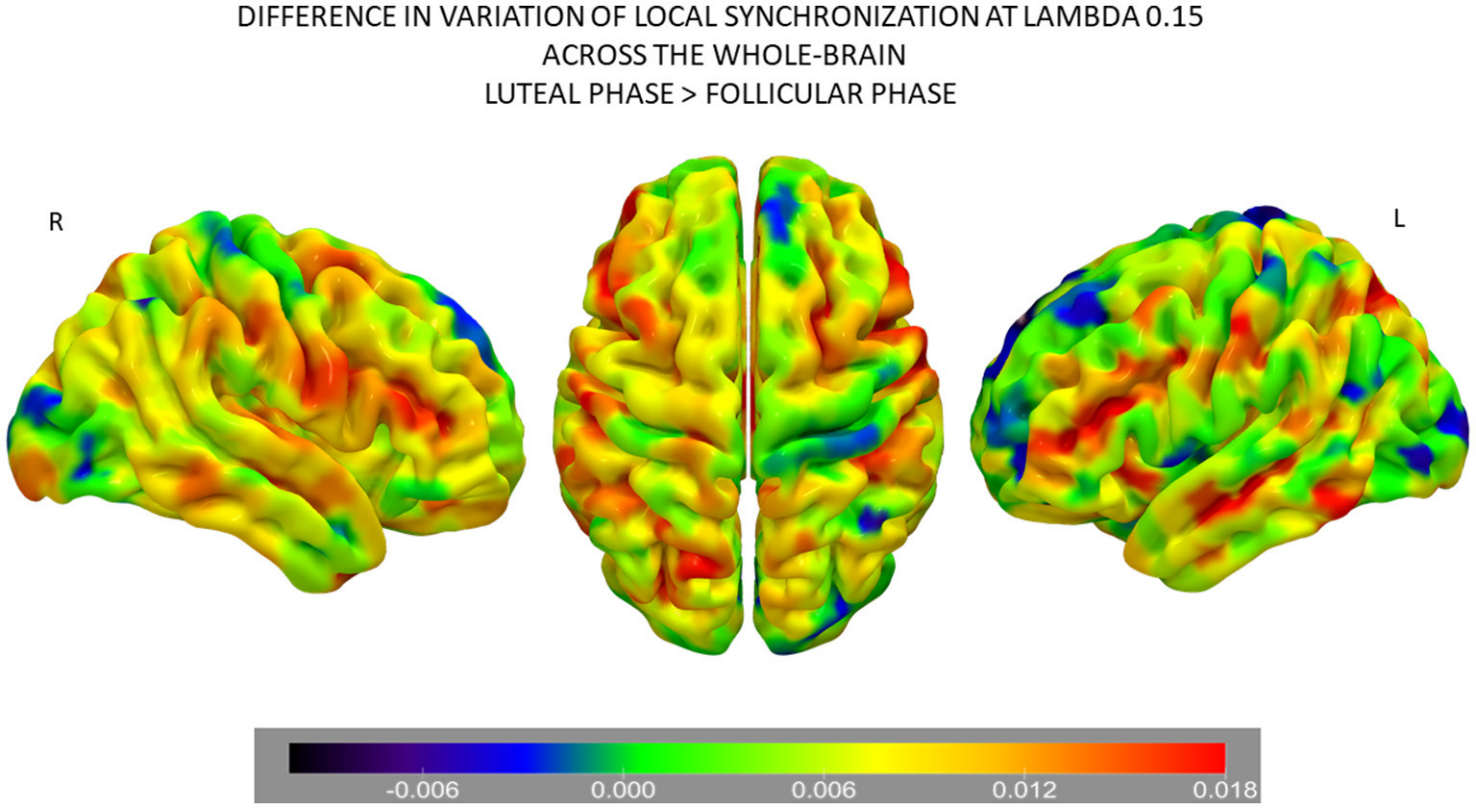
Differences in node-level information processing across the whole-brain network between the luteal and follicular phases of the NaturalMenstrualCycle Study. Rendered brains show the difference in variation of local synchronization levels for each brain area between phases. Colder colors (i.e., back-blue extreme) represent brain areas showing decreased levels of variation during the luteal phase than the follicular, while warmer colors (i.e., red extreme) mark the areas with the most significant increase in variation of local synchronization during the luteal phase. Areas marked in green are the ones showing similar variation levels across both phases. Areas belonging to the default mode, the salience/ventral attention, the somatomotor, the control, and the dorsal attention networks show the highest values of node-level synchronization during the luteal phase. The rendered brain was plotted using the Surf Ice software (https://www.nitrc.org/projects/surfice/).

Following the results of the information processing analysis, we aimed to investigate which brain areas show the highest difference between the two phases of the NaturalMenstrualCycle Study. Therefore, we calculated the difference in the variability of local synchronization across nodes between the luteal and the follicular phases (Figure 3). We found that the default mode network (DMN) was the one showing the highest increase in variation of local synchronization during the luteal phase. Furthermore, brain areas belonging to the salience/ventral attention, somatomotor, control and dorsal attention (DAN) networks also showed increased variability of local synchronization at λ = 0.15 and λ = 0.12 during the luteal phase. This result indicates that the brain's information transmission is enhanced across large-scale networks at long distances during the luteal phase.

### 3.5. Multi-Level Model With Hormone Levels and Turbulence

Finally, we explore the relationship between day-to-day hormonal concentrations when naturally-cycling (NaturalMenstrualCycle Study) and turbulence levels (i.e., where we found significant differences between phases). To this end, we carried out multi-level models for each dependent variable (turbulence at λ = 0.12 and λ = 0.15 scales) with sex hormone levels (estradiol and progesterone) as fixed effects. As random effects we included subject (*N* = 1) and testing day (observations days = 30).

**Figure 4:**
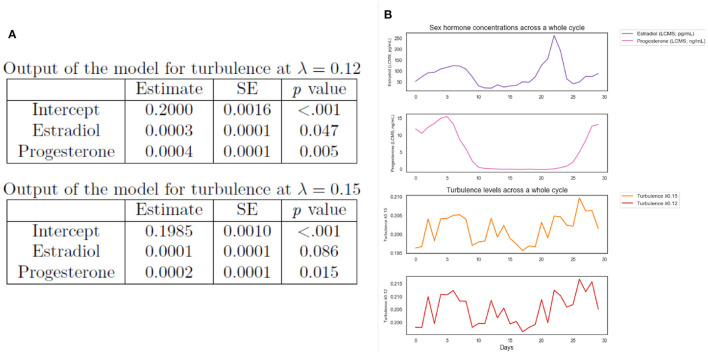
Exploratory analysis. **(A)** Multi-level models outputs of whole-brain turbulence levels predicted by day-to-day ovarian hormone concentrations for the NaturalMenstrualCycle Study. We found that estradiol and progesterone levels predicted brain turbulence at λ = 0.12. Similarly, progesterone levels predicted brain turbulence at λ = 0.15, however, estradiol was not significant at this λ scale. These results indicate that hormonal changes modulate turbulent dynamics in long distances in the brain. **(B)** Plot of estradiol (purple line) and progesterone (pink line) daily concentrations and turbulence levels at λ = 0.15 (orange line) and at λ=0.12 (red line) across the 30 days of the experiment (x-axis).

The resulting model' lmer syntax for turbulence at λ 0.12 was: *turbulence* λ *0.12* ~ *1* + *Estradiol levels + Progesterone levels* + *(1 + session* |*subject)*. The parameter estimates for the model of turbulence 0.12 showed significant main effects of estradiol (*p*=0.047) and progesterone levels (*p*=0.005). In addition, the model output for turbulence at λ = 0.15 [model syntax: *turbulence* λ *0.15* ~*1* + *Estradiol levels* + *Progesterone levels* + *(1* + *session* | *subject)*] showed the significant effect of progesterone (*p*=0.015), however, the estradiol did not show a significant effect at λ=0.15 scale (*p* = 0.086). The full outputs of the multi-level models are presented in Figure 4A. These results suggest that changes in concentrations of ovarian hormone modulate whole-brain turbulent dynamics in long distances (see Figure 4B for a visual representation of the interplay between estradiol, progesterone, and turbulence levels controlling for the experimental session).

## 4. Discussion

In the present work, we aimed to explore how the brain's information processing changes across spacetime scales between the two main phases of the menstrual cycle, namely the luteal and the follicular. Furthermore, we used a second dataset (HormonalContraceptive Study) as a comparison condition in which the same participant was on hormonal contraception. We demonstrated that information processing across scales changes significantly between the luteal and follicular phases when naturally-cycling but not under selective hormonal suppression. At the node-level, we found that the DMN, salience/ventral attention, somatomotor network, DAN, and control networks showed an increased variation of local synchronization during the luteal compared to the follicular phase. Moreover, we found that changes in estradiol and progesterone concentrations modulate whole-brain turbulent dynamics in long distances.

We showed that the luteal phase was characterized by higher turbulence levels than the follicular phase across lower spatial scales (i.e., long distances in the brain). Moreover, the analysis of information cascade flow and information cascade measures showed that efficiency in information transmission across different scales was enhanced during the luteal phase compared to the follicular. In the same way, we reported increased information transfer across the whole-brain at lower spatial scales for the luteal phase compared to the follicular when the participant was freely cycling. In contrast, we showed that being under a hormone-based regime led to a more stable pattern of turbulence levels across the whole cycle, with no significant shifts in information processing, as found for all four empirical measures of turbulent dynamics (i.e., amplitude turbulence, information cascade flow, information cascade, and information transfer).

We further computed the differences in the variability of synchronization levels across the whole-brain between the luteal and the follicular phases to highlight cycle-dependent changes in large-scale brain networks. We found that the DMN was the network presenting the most significant dynamic variation depending on the menstrual cycle phase, showing increased levels of variation in local synchronization during the luteal phase. Together with the DMN, we found that other large-scale networks such as the salience/ventral attention, somatomotor, control, and DAN showed the highest cycle-dependent changes in this measure. These results are in line with recent findings using the same dataset (Pritschet et al., 2020; Mueller et al., 2021). The work of Pritschet et al. (2020) used a time-lagged analysis and found that fluctuations in estradiol levels led to an enhancement of network efficiency, particularly for the DMN and the DAN. Accordingly, using dynamic community detection, Mueller et al. (2021) found that peaks in estradiol were reflected in a localized and transient reorganization of large-scale networks, particularly of the DMN and a control subnetwork. The authors also found that other brain networks, such as the temporoparietal, limbic, subcortical networks, showed the highest flexibility values in response to a rise in estradiol concentration (Mueller et al., 2021). Here, we expand previous findings from this dataset by demonstrating that the effects of the cycle stage were not only reflected at the spatiotemporal level of the brain's information processing but also in the transmission of information across different scales. Previous human neuroimaging studies investigating the effects of hormone fluctuation and cycle stage on the brain's functional connectivity dynamics also provided compelling evidence of hormone-related modulation of several networks (Petersen et al., 2014; Arélin et al., 2015; Lisofsky et al., 2015a; Weis et al., 2019). For example, Arélin et al. (2015), using a deep-sampling approach, demonstrated that inter-regional connectivity changed in association with progesterone levels and that increases in this sex hormone led to enhanced connectivity between the dorsolateral prefrontal cortex, hippocampus, and sensorimotor cortex. A cross-sectional study conducted by Petersen et al. (2014) found that the follicular phase compared to the luteal was associated with higher within-network connectivity for the DMN and the control network. Here, we extend these findings by demonstrating that the effects of the cycle stage are reflected in the transmission of information across different spatial scales, both for large-scale networks and across the whole-brain network.

Finally, we were interested in examining the relationship between sex hormone levels and differences in the brain's turbulence levels. Results of multi-level models controlling for the experimental session showed that estradiol and progesterone concentrations modulate information transmission across long distances in the brain when the participant was naturally-cycling. This result suggests an interplay between progesterone and estradiol with the brain's information transmission, for which variations in ovarian hormone levels alter information processing across the whole-brain network. This trend is in line with previous literature reporting an association between cortico-cortical and subcortical-cortical functional connectivity and higher concentrations of estradiol and progesterone (Peper et al., 2011). Additionally, Weis and Hausmann (2010) found that higher levels of both ovarian hormones are associated with lower interhemispheric inhibition, thus increased functional communication between the two hemispheres.

We want to highlight some limitations of the current study. We used a dense-sampling dataset from a single subject. Future studies could benefit from a dense-sampling design with a larger sample size of participants to study fine-grained phases (e.g., early- and late-follicular, ovulatory, mid-, and late-luteal).

In addition, the empirical measures applied in this study do not offer a causal explanation of cycle-dependent changes in turbulent dynamics in the human brain. A future direction would be to apply whole-brain computational modeling to shed light on the mechanisms behind sex hormone fluctuations and changes in whole-brain dynamics. A handful of studies have provided evidence of the impact of hormonal transition or suppression on the risk of developing mood disorders (Bloch et al., 2000; Young et al., 2007; Taylor et al., 2019). Therefore, elucidating the mechanisms underlying the relationship between sex hormone concentrations and brain functioning and dysfunction may foster a deeper understanding of mood disorders related to neuroendocrine change.

## 5. Conclusions

In this work, we showed how the menstrual cycle modulates whole-brain turbulent dynamics. We applied a novel turbulence framework to study whole-brain information processing across spacetime scales (i.e., over long and short distances in the brain) during both a naturally occurring menstrual cycle and under a hormonal contraceptive regime. We demonstrated that the luteal phase is characterized by higher turbulence levels at lower scales (i.e., long distances in the brain) and higher information transmission across scales. By contrast, under hormonal-based regime showed no differences in information processing across the whole menstrual cycle. Furthermore, we found that the DMN, salience/ventral attention, somatomotor, control, and dorsal attention were the large-scale networks showing the most significant increases during the luteal vs. the follicular phases. Finally, we found an interplay between progesterone and estradiol with the brain's information transmission, showing that ovarian hormone levels alter information processing across the whole-brain network. Overall, our results show that ovarian hormones and menstrual cycle stages modulate whole-brain turbulent dynamics.

## Data Availability Statement

The datasets analyzed for this study can be found in the “28andMe” open repository at https://openneuro.org/datasets/ds002674/versions/1.0.5.

## Ethics Statement

The studies involving human participants were reviewed and approved by the University of California, Santa Barbara Human Subjects Committee. The patients/participants provided their written informed consent to participate in this study. Written informed consent was obtained from the individual(s) for the publication of any potentially identifiable images or data included in this article.

## Author Contributions

GD and AE designed the study. ED, MK, YS, GD, and AE developed methodology and software. EJ, LP, and TS provided the MRI data. ED and AE preprocessed and analyzed the datasets. ED, CU, YS, and AE wrote the original draft. VG contributed to analysis and interpretation of results during the review process. All authors interpreted and discussed the results, reviewed, edited, and approved the last version of the manuscript.

## Funding

AE and GD were supported by the HBP SGA3 Human Brain Project Specific Grant Agreement 3 (grant agreement no. 945539), funded by the EU H2020 FET Flagship.

## Conflict of Interest

The authors declare that the research was conducted in the absence of any commercial or financial relationships that could be construed as a potential conflict of interest.

## Publisher's Note

All claims expressed in this article are solely those of the authors and do not necessarily represent those of their affiliated organizations, or those of the publisher, the editors and the reviewers. Any product that may be evaluated in this article, or claim that may be made by its manufacturer, is not guaranteed or endorsed by the publisher.
